# Mönckeberg's sclerosis – is the artery the only target of calcification?

**DOI:** 10.1186/1471-2261-5-34

**Published:** 2005-12-12

**Authors:** Carlos Eduardo Barra Couri, Geruza Alves da Silva, José Antônio Baddini Martinez, Francisco de Assis Pereira, Francisco José Albuquerque de Paula

**Affiliations:** 1 Department of Internal Medicine, School of Medicine of Ribeirão Preto, University of São Paulo, Ribeirão Preto, Brazil

## Abstract

**Background:**

Since its first description, Mönckeberg's sclerosis has only been related to arterial media calcification, being listed among the primary diseases of the vessels.

**Case presentation:**

We report here a clinically and histologically confirmed case of Mönckeberg's sclerosis in which the patient presented with massive areas of soft tissue calcifications in the pharynx and larynx. Polysomnographic parameters showed severe obstructive apnea refractory to nasal continuous positive airway pressure. Clinical and laboratory findings excluded concomitant endocrine or rheumatological diseases.

**Conclusion:**

Our data provide a new insight about Mönckeberg's sclerosis, i.e., the fact that the etiopathogenic process involved in the phenomenon of calcification may not be restricted only to the arteries, but may occur in the entire organism. Further studies of the etiopathogenesis of this disease are needed.

## Introduction

Mönckeberg's sclerosis (MS) is a degenerative and apparently non-inflammatory disease in which the media of small and medium-sized muscular arteries becomes calcified independently of atherosclerosis. Since the condition does not involve primarily the intimal layer of the artery, the lumen is kept open by the rigid media and, therefore, luminal narrowing is not a direct consequence [1-4]. Recent studies, however, have demonstrated that MS is a risk factor for cardiovascular disease and peripheral artery obstruction [1,4,5]. The exact etiopathogenesis of this process is far from being understood, but is frequently related to glucose intolerance, aging, male gender, autonomic neuropathy, osteoporosis and, chronic renal failure[6,7]. It commonly occurs in peripheral arteries of the lower limbs where it is seen as "rail tracking" on incidental plain radiographs[1,8].

To the best of our knowledge, soft tissue calcifications have never been described in such disease, especially in association with obstructive sleep apnea (OSA). We report here a patient with classic MS who presented severe OSA due to calcinosis of the pharynx.

## Case report

A 63-year-old Brazilian woman was referred to the hospital with a 20-year history of an increasing size of the anterior portion of the neck associated with progressive exertional dyspnea, nighttime snoring and coughing, dysphagia of solids and daytime sleepiness. She had no history of hypertension, cigarette smoking, alcohol ingestion, weight loss, fractures or fever. Her medical history was significant for intermittent claudication.

Physical examination showed a thin woman (body mass index of 17 kg/m^2^) with a hard, irregular, painless, mobile tumoration on the anterior surface of the neck. No lymph nodes were palpable and hoarseness was evident. There was no craniofacial abnormality. The results of oral cavity, thyroid, cardiopulmonary and abdominal examination were unremarkable. The femoral arteries appeared to be thickened and pulses were frail although there were no signs of acute limb ischemia.

**Figure 1:**
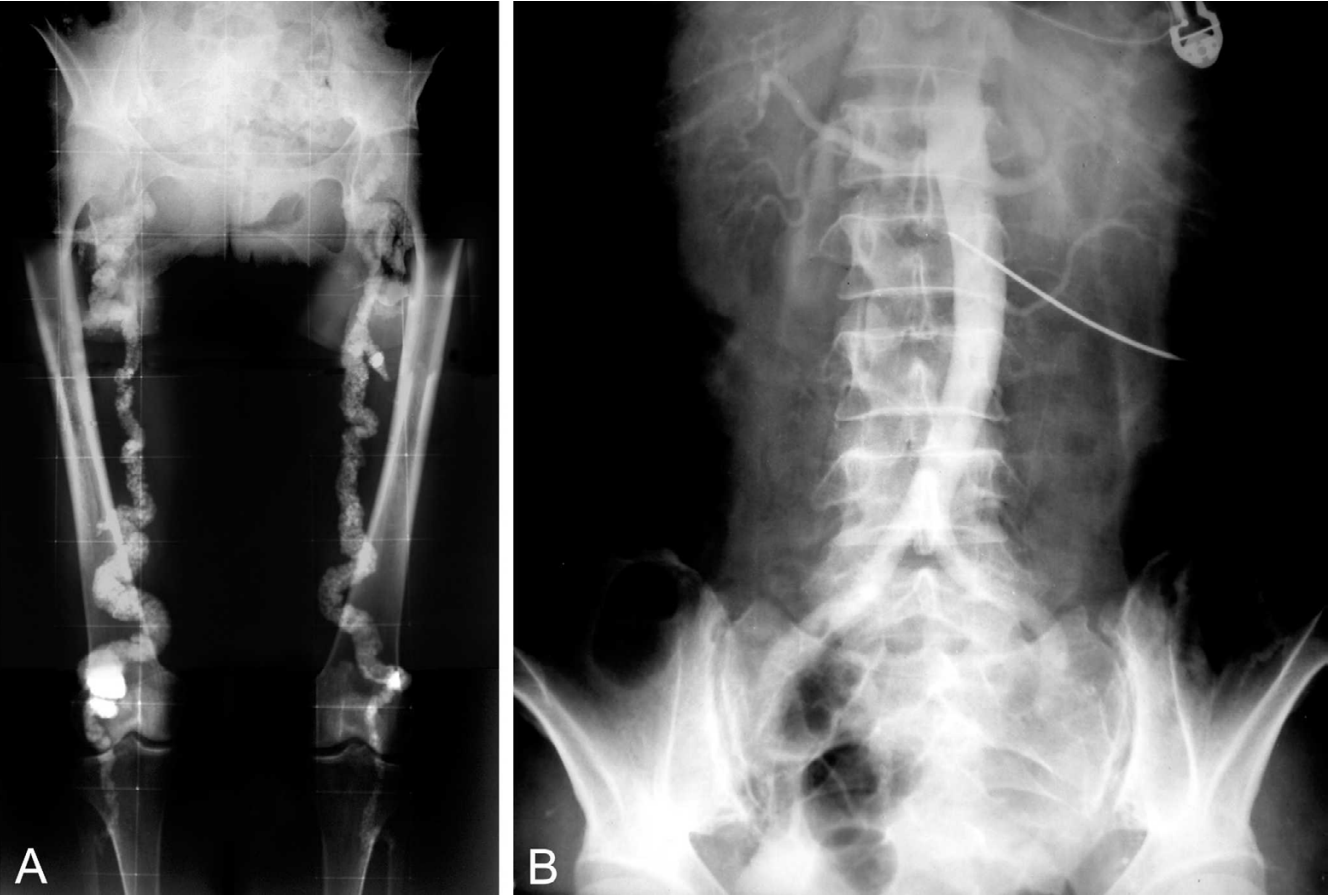
**A**. Pelvic and lower extremity radiograph shows extensive calcification of the femoral arteries. **B. **Translumbar aortography shows near-total obstruction of the femoral arteries.

**Figure 2:**
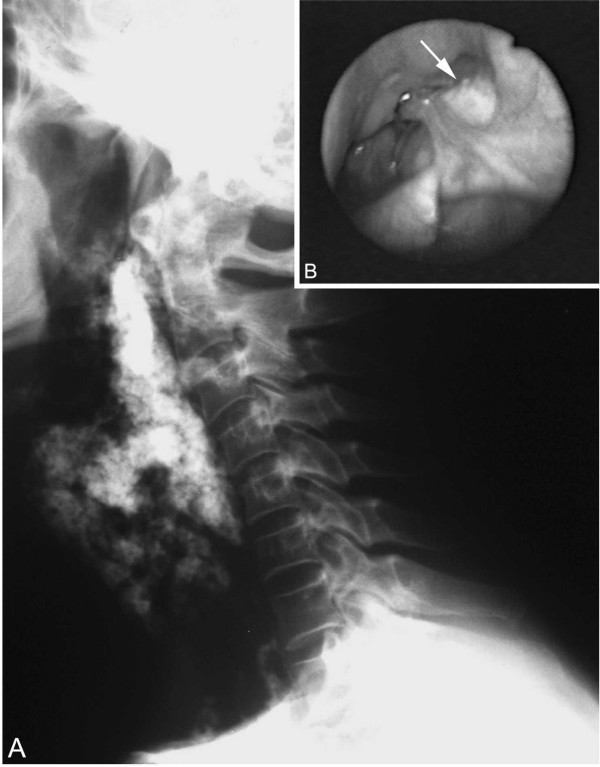
**A. **Neck radiograph evidences grossly calcified areas of soft tissue in the topography of the oropharynx. **B. **Direct laryngoscopic examination shows exophytic calcic deposits in the left pyriform sinus (arrow).

**Figure 3:**
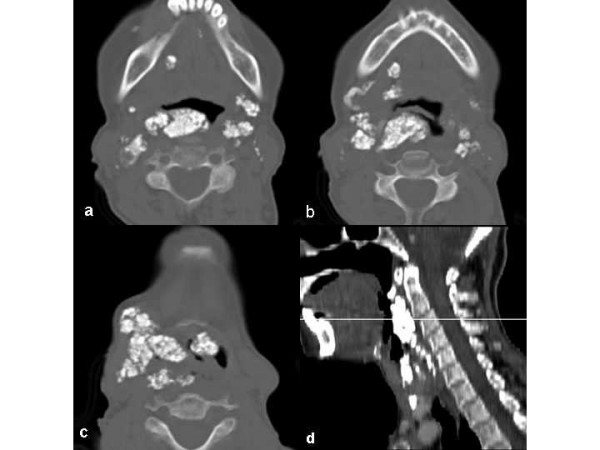
Transverse slices of CT showing multiple coarse calcifications scattered by oropharynx **(A)**, hypopharynx **(B) **and larynx **(C)**. **D. **Sagittal plane appearance.

**Figure 4:**
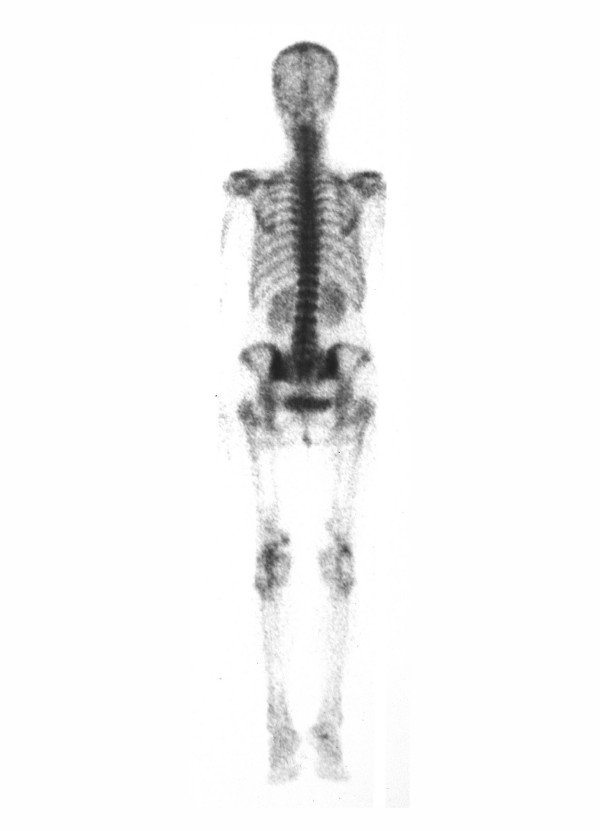
Bone scintigram shows increased uptake of ^99 m^Tc-methylene diphosphate in the calcified femoral arteries and on the anterior surface of the neck.

Pelvic and lower extremity roentgenograms showed dense, "rail tracking" femoral artery calcification associated with near-total obstruction of these vessels confirmed by translumbar aortography (figure 1). A neck X-ray and computed tomography evidenced grossly calcified areas of soft tissue in the topography of the pharynx and larynx. Direct laryngoscopy confirmed exophytic calcic deposits in the left pyriform sinus (figure 2 and 3). No other images of abnormal calcifications were found at any other sites, such as the arms, lung, heart, aorta or abdomen. A bone scintigram showed increased uptake of ^99 m^Tc-methylene diphosphate in the calcified femoral arteries and on the anterior surface of the neck (figure 4). Right hip and lumbar spine bone densitometry evidenced supportive findings of osteoporosis. Chest X-ray revealed mild enlargement of cardiac area associated with ectasic thoracic aorta; pulmonary aspects were normal. Doppler echocardiography revealed mild pulmonary hypertension (39 mmHg) associated with mild left atrium and moderate left ventricle enlargement; there was a diffuse left ventricle hypokinesia associated with an ejection fraction of 32% (indirectly measured by echocardiography). Also, there was an eccentrical left ventricular hypertrophy, severe mitral and tricuspid regurgitation. There was no abnormality in the right ventricle. Cineangiocoronariography was attempted to directly analyse any coronary disease as a possible cause of these abnormalities, but the rigidity of femoral and brachial arteries impeded the insertion of the catheter.

Sleep variables obtained from polysomnography were indicative of severe OSA (table 1) with an apnea/hypopnea index of 51.6 per hour (40.9 apnea episodes/h), 89% of which occurred together with bradycardia and extra beats. Minimal oxygen saturation was 88%. The spirometric study demonstrated mild obstructive airway disease with no responsiveness after bronchodilator test. Also, there was a moderate decrease in the pulmonary diffusion capacity (Collins GS Plus Modular System, Warren E Collins Inc. Braintree, MA).

**Table 1:** Diagnostic values of sleep and respiratory variables obtained by polysomnography

**Sleep and respiratory variables**	**Patient**	**Expected values**
Sleep efficiency (TST*/TRT† %)	58	>80
Stage 1 (%)	10,7	≤_9
Stage 2 (%)	74,4	≤_60
Stage 3–4 (%)	7,4	10–25
Stage REM (%)	7,5	10–25
Mean oxygen saturation(%)	92	>96
Minimal oxygen saturation (%)	88	93–95
Apnea/Hypopnea index (/hour)	51,6	< 5
Apnea index (/hour)	40,9	<5
Mean duration of apneas (seconds)	30	10
Time spent with SaO2 <90%(min)	2	-

Total sleep time; † Total recording time.

**Figure 5:**
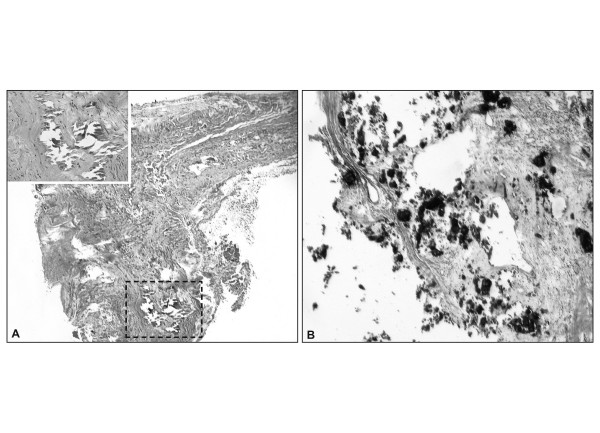
**A. **Temporal artery biopsy shows circumferential calcic deposits in the media (detail) (H&E stain). **B. **A pyriform sinus biopsy shows soft tissue calcification (arrows) (H&E stain).

Except for the impaired fasting glucose state (fasting blood glucose of 117 mg/dl), there were no other biochemical abnormalities concerning calcium, phosphate, magnesium, creatinine and parathyroid hormone. Clinical and laboratory findings excluded any kind of rheumatologic disease such as dermatomyositis, scleroderma or systemic lupus erythematosus. Biopsy specimens from the pharynx and temporal artery revealed soft tissue calcification and medial artery calcinosis, respectively (Figure 5).

Continuous positive airway pressure (CPAP) tritation for treatment of such obstructive sleep apnea has been tried twice on different occasions, but the patient has complained of extreme discomfort (she could hardly sleep during the procedures). A pressure of 10 cm H_2_O was reached during some short periods of sleep with a very low effect on the frequency of episodes of apnea. So, with the aim of stopping further vascular and soft tissue calcification, intravenous dissodic pamidronate was administred (60 mg IV diluted in 250 mL of saline solution once a day for 1 day).

## Discussion

Mönckeberg's sclerosis commonly occurs in otherwise healthy elderly patients independently of atherosclerosis. After 35 years of disease, 94% of all diabetic patients present arterial calcification[9]. This is usually an incidental finding during lower limb radiographic examination, although involvement of renal and coronary vessels has been described [3]. The cause of medial calcinosis remains unclear, but some insights into the etiopathogenic process have been provided by isolated studies. Shanahan *el al *[10] proposed that a loss of expression of certain proteins related to the inhibition of calcification could be the causative factor. These proteins are Gla protein, osteoprotegerin, fibrillin I and carbonic anhydrase (all produced by vascular smooth muscle cells). Byts *et al*. [11] pointed out that medial calcification can be a consequence of various metabolic changes triggered by a necrobiotic injury installed in the vessel wall.

Since its first description in 1903 [3], MS has only been related to media calcification of small-to-medium-sized arteries, being listed among the primary diseases of the vessels. Calcic involvement of soft tissues has never been described in MS patients. In our case the pharynx involvement was strong enough to produce severe obstructive sleep apnea and the symptoms were clearly associated with the massive tumoral growth of the anterior portion of the neck.

Obstructive sleep apnea results from repetitive episodes of pharyngeal occlusion during sleep which reverse with arousal, thus inducing sleep fragmentation, a low amount of slow wave sleep (stages 3 and 4 non-REM sleep) and a low amount of REM sleep with consequent daytime somnolence [12]. Sleep apnea causes repetitive episodes of hypoxia, hypercapnia and reoxigenation that can lead to a variety of physiological processes including pulmonary hypertension and other vascular consequences[13]. Our patient had a confirmed increase in pulmonary arterial pressure that could not be entired explained by the OSA syndrome. Probably it has been worsened by the concomitant left ventriculat disfunction. The exact cause of the cardiac disfunction could not be widely investigated, but OSA is an important contributor.

Generally, the pathogenesis of upper airway obstruction involves anatomic and neurological components. Obesity, age, male sex, positive family history and alcohol consumption are well known as major risk factors for this disorder in adults. Narrowing may occur at one or more sites in an unstable upper airway due to anatomical partial obstruction as a result of developmental delay, craniofacial abnormalities and neurologic diseases[13]. Our patient was a non-alcoholic, very slim woman (as judged by a body mass index of 17 kg/m^2^) with no family history of similar disorders.

The massive calcification areas of the pharynx were confirmed by radiological images, laryngoscopic visualization and a biopsy of the exophytic lesion in the pyriform sinus which revealed areas of soft tissue calcification. We excluded metastatic and dystrophic calcification in the light of the histological appearance of the lesion and the absence of malignancy and of rheumatologic or endocrine diseases.

A lower limb X-ray evidenced the classic "rail tracking" appearance of MS described by other authors [1,4] and a temporal artery biopsy confirmed the intense media calcinosis. The concomitant atherosclerotic process involving the intima layer of the femoral arteries explained the intermittent claudication and weak pulses presented by this patient. Both OSA and MS are recognized as causative factors of atherosclerosis[1,13]. Additionally, our patient presented low bone mineral density, and this finding supports recent evidences showing association between arterial calcification and osteoporosis[14,15].

As can be seen in figure 3, the amount of airway obstruction caused by the grossly calcified tissue could be the answer for the discomfort related to CPAP titration. In face of the difficulties related to this approach, tracheostomy was thought to solve this problem. However, the risk of triggering even more tissue calcification secondary to tissue damages discouraged the realization of this procedure.

Alternatively, treatment with intravenous dissodic pamidronate was attempted to stop the phenomenon of calcification and prevent further airway obstruction. This drug has the property to attach to hydroxyapatite crystals preventing both vascular and soft tissue calcification[16]. Additionally, it worked as a treatment for osteoporosis presented by the patient.

Although one can argue about the possible role of upper airway obstruction in intensifying the local deposition of calcium in the soft tissue of the pharynx, the diagnosis of MS was well documented in this case. The present report provides a new insight about this disease, i.e., the fact that the etiopathogenic process involved in the phenomenon of calcification may not be restricted only to the arteries, but may involve the entire organism.

## Conclusion

Monckeberg's sclerosis is a primary disease of the arteries. However, the present case report raises extensive questions about the etiopathogenic process involved in MS, since this is the first report of soft tissue calcification related to this disease. Further investigations of the etiology and pathogenesis of this disease are needed.

## Competing interests

The author(s) declare that they have no competing interests.

## Authors' contributions

GAS and JABM were responsible for the initial evaluation and management of the patient. GAS was also responsible for the realization and interpretation of polysomnography. CEBC, FAP and FJAP were the responsible for the investigation and the diagnosis of Mönckeberg's sclerosis. All authors have equally contributed in the preparation and revision of the manuscript. All authors read and approved the final manuscript.

## Pre-publication history

The pre-publication history for this paper can be accessed here:


